# Effects of combined treatment with rapamycin and cotylenin A, a novel differentiation-inducing agent, on human breast carcinoma MCF-7 cells and xenografts

**DOI:** 10.1186/bcr1344

**Published:** 2005-11-09

**Authors:** Takashi Kasukabe, Junko Okabe-Kado, Nobuo Kato, Takeshi Sassa, Yoshio Honma

**Affiliations:** 1 Research Institute for Clinical Oncology, Saitama Cancer Center, Saitama, Japan; 2 The Institute of Scientific and Industrial Research, Osaka University, Osaka, Japan; 3 Department of Bioresource Engineering, Yamagata University, Tsuruoka, Japan; 4 School of Medicine, Shimane University, Izumo, Japan

## Abstract

**Introduction:**

Rapamycin, an inhibitor of the serine/threonine kinase target of rapamycin, induces G_1 _arrest and/or apoptosis. Although rapamycin and its analogues are attractive candidates for cancer therapy, their sensitivities with respect to growth inhibition differ markedly among various cancer cells. Using human breast carcinoma cell line MCF-7 as an experimental model system, we examined the growth-inhibitory effects of combinations of various agents and rapamycin to find the agent that most potently enhances the growth-inhibitory effect of rapamycin.

**Method:**

We evaluated the growth-inhibitory effect of rapamycin plus various agents, including cotylenin A (a novel inducer of differentiation of myeloid leukaemia cells) to MCF-7 cells, using either MTT assay or trypan blue dye exclusion test. The cell cycle was analyzed using propidium iodide-stained nuclei. Expressions of several genes in MCF-7 cells with rapamycin plus cotylenin A were studied using cDNA microarray analysis and RT-PCR. The *in vitro *results of MCF-7 cells treated with rapamycin plus cotylenin A were further confirmed *in vivo *in a mouse xenograft model.

**Results:**

We found that the sensitivity of rapamycin to MCF-7 cells was markedly affected by cotylenin A. This treatment induced growth arrest of the cells at the G_1 _phase, rather than apoptosis, and induced senescence-associated β-galactosidase activity. We examined the gene expression profiles associated with exposure to rapamycin and cotylenin A using cDNA microarrays. We found that expressions of cyclin G_2_, transforming growth factor-β-induced 68 kDa protein, BCL2-interacting killer, and growth factor receptor-bound 7 were markedly induced in MCF-7 cells treated with rapamycin plus cotylenin A. Furthermore, combined treatment with rapamycin and cotylenin A significantly inhibited the growth of MCF-7 cells as xenografts, without apparent adverse effects.

**Conclusion:**

Rapamycin and cotylenin A cooperatively induced growth arrest in breast carcinoma MCF-7 cells *in vitro*, and treatment with rapamycin and cotylenin A combined more strongly inhibited the growth of MCF-7 cells as xenografts *in vivo *than treatment with rapamycin or cotylenin A alone, suggesting that this combination may have therapeutic value in treating breast cancer. We also identified several genes that were markedly modulated in MCF-7 cells treated with rapamycin plus cotylenin A.

## Introduction

Breast cancer is the most frequent cancer disease among women in the Western world, accounting for almost 30% of all cancers among women. Although there have been advances in the areas of early detection and treatment, the incidence of this disease has increased and mortality rates are almost unaltered [1]. Because oestrogen exposure is considered to be a major factor in the development of breast cancer and because most breast cancers maintain their hormonal dependency, the nonsteroidal antioestrogen tamoxifen has been the leading drug in the treatment of advanced breast cancer for more than 30 years. However, the development of resistance to antihormonal therapy is a major problem in the treatment of breast cancer patients [2,3]. Therefore, the development of a new strategy for suppressing the growth of breast cancer cells is required.

Rapamycin and its analogues are promising new drugs that use alternative mechanisms to inhibit the growth of breast cancer cells [4,5]. Rapamycin, a macrolide fungicide, was first isolated from *Streptomyces hygroscopicus *in the early 1970s and was initially developed clinically for its immunosuppressant properties. Subsequently, rapamycin became of significant interest as a potential antitumour drug.

Rapamycin first binds the 12-kDa immunophilin FK506-binding protein (FKBP12), and this complex then inhibits mammalian target of rapamycin (mTOR) – a serine/threonine kinase. mTOR is recognized as a central controller of eukaryotic cell growth and proliferation, in that it senses nutritional status and mitogens in mammalian cells and allows for the progression from G_1 _to S phase, although it may not be the only target of rapamycin. Clinically, rapamycin analogues with improved stability and pharmacological properties have been well tolerated by patients in phase I trials, and these agents have exhibited a promising antitumour effect in several types of refractory tumour, including breast cancer [6,7]. However, the sensitivities of rapamycin with respect to growth inhibition differ markedly among various cancer cells, and only a minority of patients in each tumour lineage appear to respond to rapamycin analogues [5]. To improve therapeutic efficacy against a broad range of human tumour cells, we must develop new and potent derivatives of rapamycin. Alternatively, application of synergistic combinations of rapamycin and some agents may lead to a potent therapy for some types of solid tumours.

Differentiation-inducing agents can alter the phenotype of cancer cells, including their sensitivity to anticancer drugs. We previously reported that treatment with hemin, an inducer of erythroid differentiation, greatly increased the sensitivity of human myeloid leukaemia K562 cells to 1-β-d-arabinofuranosylcytosine, and that erythroid differentiation factor (activin A) enhanced the sensitivity of multidrug-resistant leukaemia cells to vincristine, actinomycin D and doxorubicin [8,9]. In the present investigation, we examined the synergistic effects of various differentiation-inducing agents and rapamycin on the growth of mammary carcinoma cells to identify the most potent and clinically applicable drugs. The most effective agent was cotylenin A (CN-A), which has a novel fusicoccane-diterpene glycoside with a complex sugar moiety. It was originally isolated as a plant growth regulator, and has been shown to affect several physiological processes in higher plants and to have differentiation-inducing activity in several human and murine myeloid cell lines [10-14]. In leukaemia cells that were freshly isolated from patients with acute myelogenous leukaemia, CN-A has also been found to affect the differentiation of cells in primary culture [15]. This differentiation-inducing activity was more potent than those of all-*trans *retinoic acid and 1α,25-dihydroxyvitamin D_3_.

Apart from the potent differentiation-inducing activity *in vitro*, the administration of CN-A also significantly prolonged the survival of mice with severe combined immunodeficiency that had been inoculated with cells of human promyelocytic leukaemia cell line NB4 [16]. No appreciable adverse effects were observed with this treatment, suggesting that CN-A may be useful in treating leukaemia and other malignancies.

Recently, we found that CN-A and interferon-α synergistically inhibited growth and induced apoptosis in several human non-small-cell lung carcinoma cell lines. Furthermore, this combined treatment markedly inhibited the growth of human lung cancer cells as xenografts [17]. In the present study, we found that treatment with the combination of rapamycin and CN-A synergistically inhibited the proliferation of human breast cancer MCF-7 cells *in vitro*, and that this combined treatment also induced growth arrest of the cells at G_1 _phase, rather than inducing apoptosis. We also identified several genes that were markedly modulated in MCF-7 cells treated with rapamycin plus CN-A. Furthermore, we examined the therapeutic effects on xenografts of human breast carcinoma cells.

## Materials and methods

### Materials

Rapamycin was purchased from Sigma Chemical (St. Louis, MO, USA). CN-A was purified from a stock ethyl acetate extract obtained from the culture filtrate of *Cladosporium *fungus sp. 501-7W by flash chromatography on silica gel with more than 99% purity [10,11]. A stock solution of CN-A was prepared in absolute ethanol at 20 mg/ml.

### Cells and cell culture

Human breast carcinoma cell lines (MCF-7, T-47D and MDA-MB-231) and human promyeloblastic leukaemia cell line NB-4 were cultured in RPMI 1640 supplemented with 10% foetal bovine serum at 37°C in a humidified atmosphere of 5% carbon dioxide in air.

### Assay of cell growth

The cells were seeded at 1–3 × 10^4^/ml in a 24-well multidish. After culture with or without the test compounds for the indicated times, viable cells were examined using either the trypan blue dye exclusion test or a modified MTT (3-(4,5-dimethylthiazol-2-yl)-2,5-diphenyltetrazolium bromide) assay. In the MTT assay, 100 μl MTT solution (1 mg/ml in PBS) was added to each well and cells were incubated for 4 hours. The cells were then centrifuged at 1000 *g *for 10 min and the precipitates were dissolved in 1 ml dimethyl sulphoxide; their absorption at 560 nm was determined. Assay of the cumulative cell number was determined as described elsewhere [17].

### Analysis of the effects of combinations of drugs

Isobologram analysis was used to determine the effects of combinations of drugs on MCF-7 cells. Dose-dependent effects were determined for each compound and for one compound with fixed concentrations of another. The interaction of two compounds was quantified by determining the combination index (CI), in accordance with the following classic isobologram [18]:

CI = (D)_1_/(Dx)_1 _+ (D)_2_/(Dx)_2_

Where Dx is the dose of one drug alone required to produce an effect, and (D)_1 _and (D)_2 _are the doses of compounds 1 and 2, respectively, in combination that produce the same effect. From this analysis, the combined effects of the two drugs can be summarized as follows: CI = 1 indicates summation (additive and zero interaction); CI < 1 indicates synergism; and CI > 1 indicates antagonism.

### Cell cycle analysis

The cell cycle was analyzed using propidium iodide-stained nuclei. Samples of 2 × 10^6 ^cells were harvested at the time points indicated, washed in ice-cold PBS, fixed by the addition of 100% ethanol and left for 30 min on ice. The cell pellet was washed and suspended in 200 μl 1.12% sodium citrate containing RNase A (250 μg/ml) for 30 min at room temperature. Thereafter, the cells were stained with 50 μg/ml propidium iodide in the presence of 1.12% sodium citrate and analyzed in a fluorescence-activated cell sorter.

### Assay of E-cadherin and senescence markers

The expression of E-cadherin was detected by immunocytochemistry. Cells were fixed in 4% paraformaldehyde in PBS and permeabilized in acetone at 4°C. Slides were pretreated in PBS containing 0.2% Tween-20, blocked with 5% normal goat serum and 0.2% Tween-20 in PBS, and incubated with rabbit polyclonal antibody to E-cadherin (Santa Cruz, CA, USA). Antibody–antigen complexes were visualized using DAKO ENVISION/AP kit (DakoCytomation Japan, Kyoto, Japan). Senescence-associated β-galactosidase (SA-βGal) activity was determined as described by Dimri and coworkers [19]. SA-βGal activity was monitored visually by scoring blue precipitate in the cytoplasm.

### cDNA microarray analysis

Total RNA was isolated from MCF-7 cells treated with or without compound for 12 hours using Isogen (Nippon Gene, Toyama, Japan) [20]. Poly(A)^+^RNA was reverse transcribed with the concomitant incorporation of Cy3- and Cy5-labelled nucleotides. The labelled probes were hybridized with a cDNA microarray, representing about 16,000 different human genes (TaKaRa Bio Inc., Tokyo, Japan), and their fluorescent intensities were scanned according to the protocol standardized by TaKaRa Bio Inc. The genes were screened by analyzing the difference in expression profiles between two genes.

### Gene expression analysis by RT-PCR

Total RNA was extracted using Isogen (Nippon Gene), in accordance with the manufacturer's instructions. Total RNA (1 μg) from tumour cells was converted to first-strand cDNA primed with random nonamer in a reaction volume of 20 μl using an RNA PCR kit (TaKaRa Bio), and 4 μl of this reaction was used as a template in the PCR. The oligonucleotides used in PCR amplification are summarized in Table 1. PCR consisted of 25 cycles for transforming growth factor-β-induced 68 kDa protein (TGFBI), BCL2-interacting killer (BIK) and early growth response (EGR)3; 22 cycles for cyclin G_2_; 27 cycles for growth factor receptor-bound (GRB)7; and 17 cycles for glyceraldehyde-3-phosphate dehydrogenase (GAPDH); with denaturing at 94°C for 30 s, annealing at 60°C for 30 s and extension at 72°C for 30 s. The linearity of the quantitation of RT-PCR products was determined using [α-^32^P]dCTP and various amounts of total RNA, as described in the literature [20]. Under these conditions, the amounts of PCR products increased linearly up to 0.4 μg total RNA.

### Transplantation of MCF-7 cells into nude mice and treatment

Female athymic nude mice with a BALB/c genetic background were supplied by CLEA Japan (Tokyo, Japan). They were housed under specific pathogen-free conditions. The *in vivo *experiments were performed in accordance with the guidelines of our institute (Guide for Animal Experimentation, Saitama Cancer Center, Saitama, Japan). One week before MCF-7 cell inoculation, mice each received 2 μg of 17β-oestradiol valerate (Sigma), dissolved in 0.2 ml of sesame oil, by subcutaneous injection. Oestrogen injections were repeated every week to sustain tumour growth. Mice were inoculated subcutaneously with 2 × 10^7 ^MCF-7 cells. By day 21 after inoculation, all of the tumours were about the same size. The animals were randomly distributed into four groups of 20 mice each. A stock solution of CN-A for administration was prepared in dimethyl sulphoxide at 100 mg/ml, and rapamycin was dissolved in ethanol at 1 mg/ml. Mice were given a daily intraperitoneal injection of 0.1 ml PBS, including 100 ng rapamycin, and/or subcutaneous injections every other day of 0.2 ml of PBS, including 100 μg CN-A, at a site distant from the tumours. Tumour size was measured with vernier calipers every other day. Statistical analysis was performed using Student's t-test.

## Results

### Combined effects of rapamycin and cotylenin A on the growth of MCF-7 cells

We examined the synergistic effects of various agents, including differentiation inducers and rapamycin, on growth inhibition of cells of breast cancer cell line MCF-7. Sensitivity to anticancer agents such as 5-fluorouracil, cisplatin and doxorubicin was barely affected by rapamycin. All-*trans *retinoic acid (a typical differentiation inducer), but not vitamin D_3_, affected the growth of MCF-7 cells, and the growth-inhibitory effect of rapamycin was significantly enhanced by all-*trans *retinoic acid, although a high concentration of retinoic acid was required (data not shown).

**Figure 1:**
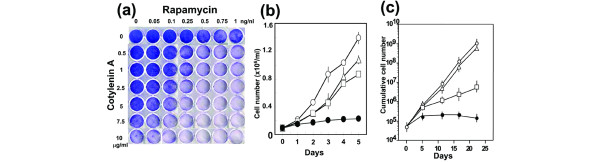
Synergistic effects of rapamycin and CN-A on the growth of MCF-7 cells. **(a) **Cells (3 × 10^4 ^cells/ml) were cultured with various concentrations of rapamycin and CN-A for 5 days. After culture, cells were stained with May–Grunwald–Giemsa. **(b) **Cells (3 × 10^4 ^cells/ml) were cultured without (white circle) or with (white triangle) 0.5 ng/ml rapamycin, 10 μg/ml CN-A (white square), or 0.5 ng/ml rapamycin plus 10 μg/ml CN-A (black circle) for the indicated number of days. The values are expressed as mean ± standard deviation of four determinations. **(c) **Cells were cultured without (white circle) or with (white triangle) 0.5 ng/ml rapamycin, 10 μg/ml CN-A (white square), or 0.5 ng/ml rapamycin plus 10 μg/ml CN-A (black circle) for the indicated number of days. The culture medium was replaced by fresh medium at least once every 5 days. The cell density was kept at 1–8 × 10^4 ^cells/ml. The values are expressed as mean ± SD of four separate experiments. CN-A, cotylenin A.

**Figure 2:**
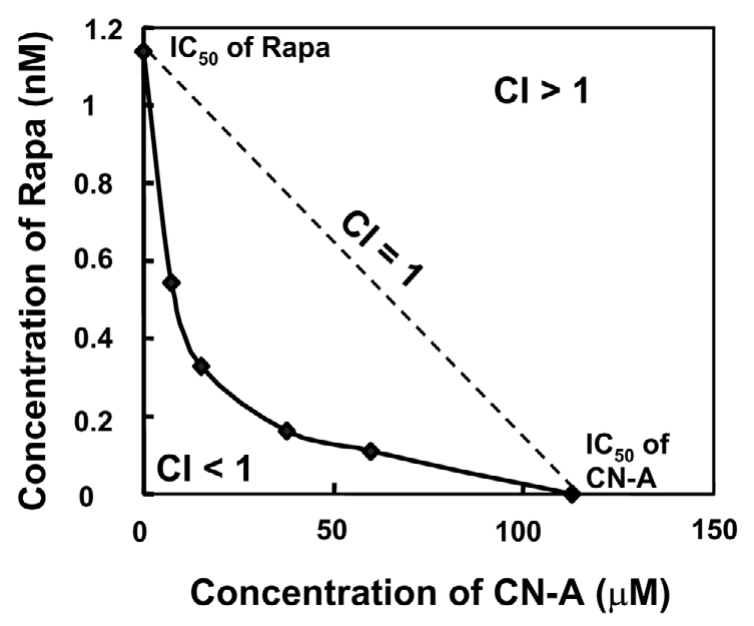
Classic isobologram at IC_50_: rapamycin plus CN-A in MCF-7 cells. Isoboles for the combination of rapamycin with CN-A that were isoeffective (IC_50_) for inhibition of proliferation of MCF-7 cells. The dashed line indicates the zero interaction of the isobole. Cells (3 × 10^4 ^cells/ml) were treated with rapamycin and CN-A for 5 days. Values are the means of three separate experiments. CN-A, cotylenin A; IC_50_, the concentration of the drug required for 50 % inhibition of cell growth.

Among the differentiation-inducing agents tested, the sensitivity of MCF-7 cells to rapamycin was most affected by CN-A, a novel inducer of the differentiation of myeloid leukaemia [12-16]. Figure 1 shows the synergistic effects of rapamycin and CN-A on the growth of MCF-7 cells. Figure 1a shows the May–Gruenwald–Giemsa staining pattern of MCF-7 cells after 5-days of treatment with various concentrations of both rapamycin and CN-A. Figure 1b shows the time course of the combined effects of rapamycin and CN-A on the growth of MCF-7 cells. The growth of MCF-7 cells was inhibited moderately by rapamycin (0.5 ng/ml) or CN-A (10 μg/ml) alone, but growth was still seen at least until 5 days, whereas the cell number did not change after 1 day of treatment with the combination of rapamycin and CN-A. Figure 2 shows isoboles for the combination of rapamycin with CN-A that were isoeffective (IC_50 _: the concentration of the drug required for 50 % inhibition of cell growth) for inhibition of proliferation of MCF-7 cells. These isoboles indicate that the combination of these drugs had synergistic effects.

We also examined the long-term effects of combined treatment with rapamycin and CN-A on the proliferation of MCF-7 cells (Fig. 1c). The growth rate of rapamycin-treated cells was almost the same as that of control cells after 5 days. Cell growth was significantly inhibited by CN-A alone after 5 days, but the cell number was still increased even at day 23. On the other hand, cell growth was greatly inhibited by combined treatment with rapamycin and CN-A, and the cell number at day 23 was almost the same as that at day 5 (Fig. 1c).

**Figure 3:**
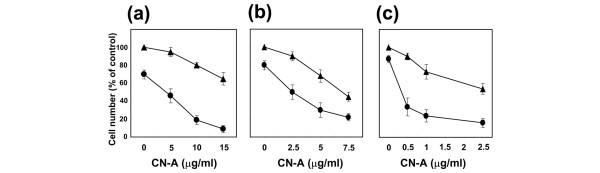
Combined effect of rapamycin and CN-A on the growth of human cancer cells. **(a) **human breast cancer T-47D cells (3 × 10^4 ^cells/ml) were treated with CN-A in the absence (black triangle) or the presence (black circle) of 0.5 ng/ml rapamycin for 5 days. **(b) **human breast cancer MBA-MB-231 (3 × 10^4 ^cells/ml) cells were treated with CN-A in the absence (black triangle) or the presence (black circle) of 15 ng/ml rapamycin for 5 days. **(c) **human promyelocytic leukaemia NB-4 cells (3 × 10^4 ^cells/ml) were treated with CN-A in the absence (black triangle) or the presence (black circle) of 0.5 ng/ml rapamycin for 5 days. Values are expressed as mean ± standard deviation for three separate experiments. CN-A, cotylenin A.

Similar growth-inhibitory effects of rapamycin and CN-A were observed in two other human breast cancer cell lines (oestrogen receptor-positive T-47D cells and oestrogen receptor-negative MDA-MB-231 cells; Fig. 3a,b). Furthermore, these combined effects of rapamycin and CN-A were also observed in several different tumour cell lines such as human promyelocytic leukaemia NB-4 cells (Fig. 3c) and human non-small-cell lung carcinoma A549 and Lu99 cells (data not shown). These findings indicate that the growth-inhibitory effects of rapamycin plus CN-A were not restricted to MCF-7 cells.

To establish the most effective treatment procedure using rapamycin and CN-A on MCF-7 cells, we examined the effects of various different treatments on MCF-7 cells. MCF-7 cells were seeded at 1 × 10^4 ^cells/ml and the number of viable cells was determined by MTT assay after 7 days. Simultaneous treatment with rapamycin (0.5 ng/ml) and CN-A (2.5 μg/ml) for 7 days was most effective at inhibiting cell proliferation (96% inhibition). Furthermore, we found that the timing of the addition of CN-A was critical to the inhibitory affect on the cell number. When CN-A was added at day 2, the combined growth-inhibitory effect was markedly decreased at 7 days (29% inhibition). On the other hand, when rapamycin was added at day 1 or day 2, the combined growth-inhibitory effects were almost completely conserved at 7 days (95% or 90% inhibition, respectively). We also found that an initial short exposure to CN-A was enough to exert the combined effect. When cells were treated with CN-A for only 1 day from day 0 to day 1 in the presence of rapamycin, and then cultured only in the presence of rapamycin from day 2 to day 7, the combined growth-inhibitory effects were still almost completely preserved at 7 days (90% inhibition). These findings indicate that simultaneous treatment with rapamycin and CN-A is the most effective procedure for inhibiting the cell growth of MCF-7 cells and that initial 1-day treatment with CN-A and continuous treatment with rapamycin has almost the same growth-inhibitory effects as simultaneous treatment with rapamycin and CN-A.

### Induction of G_1 _arrest in MCF-7 cells treated with rapamycin plus cotylenin A

**Figure 4:**
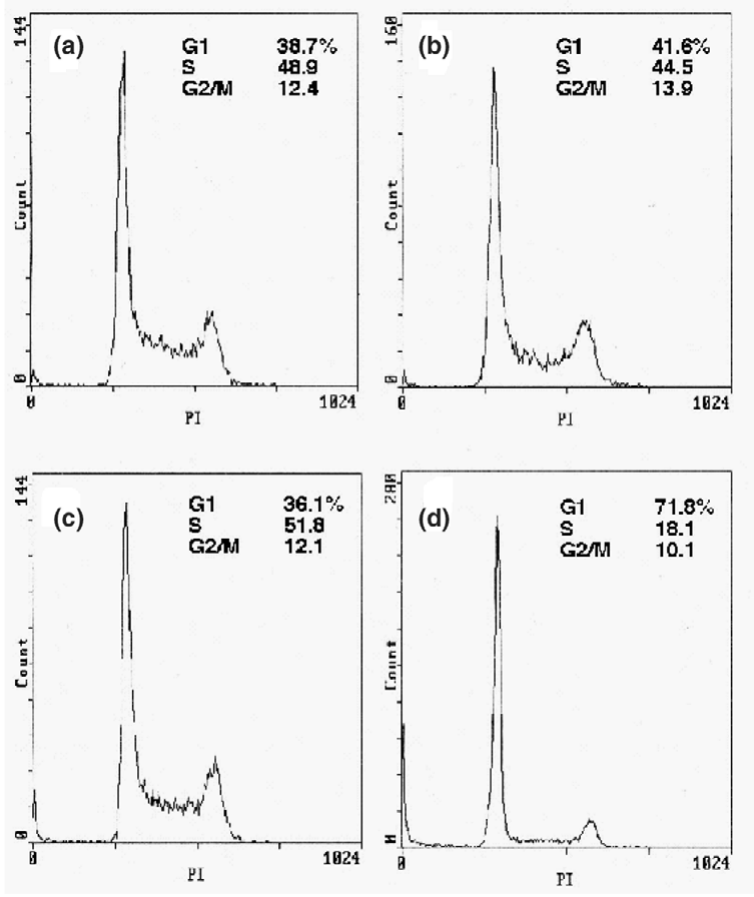
Induction of G_1 _arrest in MCF-7 cells treated with rapamycin plus CN-A. Cells were cultured **(a) **without or **(b) **with 10 μg/ml CN-A, **(c) **0.5 ng/ml rapamycin, or **(d) **0.5 ng/ml rapamycin plus 10 μg/ml CN-A for 6 days, and DNA histograms were then analyzed. CN-A, cotylenin A.

To better understand the combined effects of rapamycin and CN-A on cell growth, we exposed MCF-7 cells to 0.5 ng/ml rapamycin and 10 μg/ml CN-A, and then measured the changes in the cell cycle distribution after 6 days. This concentration of rapamycin or CN-A alone did not affect the cell cycle in our study, whereas rapamycin with CN-A induced growth arrest of cells at the G_1 _phase (Fig. 4). The induction of apoptosis (cells in sub-G_1 _phase) was not observed in cells with the combined treatment.

### Induction of phenotypic changes in MCF-7 cells treated with rapamycin and cotylenin A

**Figure 5:**
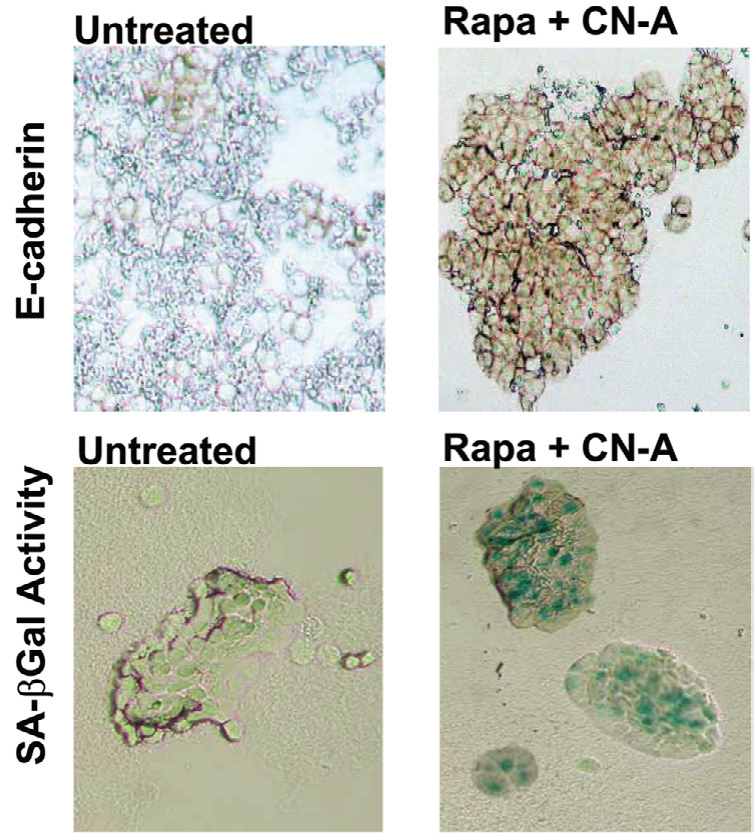
Induction of phenotypic changes in MCF-7 cells treated with CN-A and rapamycin. Upper row: E-cadherin. Cells were cultured in the presence (right) or absence (left) of 10 μg/ml CN-A plus 0.5 ng/ml of rapamycin for 7 days. Lower row: SA-βGal activity. Cells were cultured in the presence (right) or absence (left) of 10 μg/ml CN-A plus 0.5 ng/ml rapamycin for 7 days. CN-A, cotylenin A; Rapa, rapamycin; SA-βGal, senescence-associated β-galactosidase.

Because treatment with rapamycin and CN-A induced G_1 _arrest but not apoptosis, these treatments may induce phenotypic changes in MCF-7 cells. Changes in several differentiation-associated phenotypes were examined, but the expression of casein or mucin was not significantly induced by the combined treatment. Intracellular accumulation of lipid droplets was also not observed (data not shown). However, when we cultured MCF-7 cells for 7 days, CN-A greatly enhanced the expression of E-cadherin, and the expression was significantly enhanced by rapamycin (Fig. 5). We also found that CN-A alone or CN-A plus rapamycin could induce activity of SA-βGal (Fig. 5), which is one of the markers of senescence of epithelial cells [21]. These findings suggest that this combined treatment with rapamycin and CN-A induced growth arrest of cells at the G_1 _phase and may induce phenotypic changes toward cell senescence but not apoptosis.

### Effects of the combined treatment with rapamycin plus cotylenin A on expression of p53, p21^Cip1^, p27^Kip1 ^and cyclins

**Figure 6:**
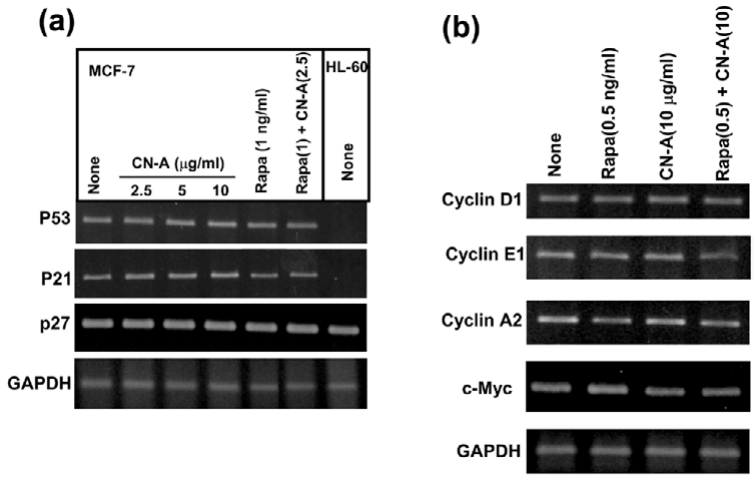
Effects of rapamycin and CN-A on the expressions of p53, p21^Cip1^, p27^Kip1 ^and cyclins. Expression of mRNAs was examined by RT-PCR analysis. **(a) **MCF-7 cells were cultured with 2.5–10 μg/ml CN-A or 1 ng/ml rapamycin, or both combined for 24 hours. **(b) **MCF-7 cells were cultured with 0.5 ng/ml rapamycin or 10 μg/ml CN-A, or both combined for 12 hours. The levels of GAPDH expression are shown to demonstrate that equal amounts of RNA were used for RT-PCR. CN-A, cotylenin A; GAPDH, glyceraldehyde-3-phosphate dehydrogenase.

To elucidate the mechanism underlying the G_1 _arrest mediated by rapamycin plus CN-A, we then examined the expression of several genes, using RT-PCR, that are important for cell cycle regulation. We first examined the expression of p53 in MCF-7 cells (Fig. 6a). Unexpectedly, the expression of p53 was not affected when MCF-7 cells were treated with 1 ng/ml rapamycin alone, 2.5–10 μg/ml CN-A alone, or 1 ng/ml rapamycin plus 2.5 μg/ml CN-A for 24 hours. HL60 cells were used as a negative control for p53 expression.

Because the p21^Cip1 ^and p27^Kip1 ^Cdk inhibitors play important roles in cell cycle regulation, especially in response to external agents, we then examined the levels of expression of these two genes (Fig. 6a). The expression levels of p21^Cip1^, which is a target gene of p53, and p27^Kip1 ^were also not induced by these treatments. Cyclin D_1 _plays an important role in G_1 _phase cell cycle progression, and previous work demonstrated that rapamycin decreased expression of cyclin D_1 _and c-myc [22]. When MCF-7 cells were treated with 0.5 ng/ml rapamycin and 10 μg/ml CN-A for 12 hours, expression of cyclin D_1 _or c-myc was not inhibited by the combined treatments (Fig. 6b). On the other hand, expression of cyclin E_1 _was slightly inhibited by the combined treatments (Fig. 6b).

### cDNA microarray analysis of MCF-7 cells treated with rapamycin, cotylenin A, and rapamycin + cotylenin A

Because we could not detect major changes in the expression of cell cycle regulating genes using RT-PCR (Fig. 6), we conducted a cDNA microarray analysis of MCF-7 cells and screened genes that were upregulated or downregulated by rapamycin plus CN-A. MCF-7 cells were cultured with 0.5 ng/ml rapamycin or 10 μg/ml CN-A, or both combined for 12 hours and total RNA was used in the microarray analysis. When MCF-7 cells were cultured with the same treatments for 5-day, the levels of growth inhibition induced by rapamycin, CN-A, and rapamycin plus CN-A were 31%, 40% and 90%, respectively.

Genes with a treatment:control ratio greater than 2 were considered to be significantly upregulated, and those with a ratio below 0.5 were considered to be significantly downregulated. Many more genes were upregulated and downregulated upon exposure to rapamycin plus CN-A than with rapamycin or CN-A alone. Whereas individually rapamycin and CN-A upregulated 61 and 46 genes and downregulated 33 and 26 genes, respectively, combined treatment with rapamycin and CN-A upregulated 225 genes and downregulated 186 genes. Furthermore, 41 genes were upregulated more than threefold in MCF-7 cells treated with rapamycin plus CN-A, although only one gene and eight genes were upregulated more than threefold in rapamycin-treated and CN-A-treated MCF-7 cells, respectively. These findings suggest that rapamycin and CN-A can also induce synergistic effects on the induction of gene expression. Table 2 shows 25 genes that were highly upregulated (>3.4-fold) by rapamycin plus CN-A. Among these 25 upregulated genes, nine and 17 were upregulated (>2-fold) in rapamycin-treated and CN-A-treated cells, respectively. TGFBI [23], BIK [24], cyclin G_2 _[25,26] and GRB7 [27] were upregulated by more than 5.8-fold in MCF-7 cells treated with rapamycin plus CN-A.

Table 3 shows 25 genes that were highly downregulated (gene expression ratio <0.38) by both rapamycin and CN-A. Thirteen genes were downregulated by less than one-third in cells treated with the combined treatment. In cells treated with CN-A alone or the combined treatment, the expression of EGR3 [28] was decreased to about one-fifth of that in control MCF-7 cells. Table 4 shows the effects of rapamycin plus CN-A on the expressions of several cell cycle regulatory genes. As mentioned above, the expression of cyclin G_2 _(gene expression ratio 6.08) was markedly induced, whereas the expressions of cyclin E_1 _(gene expression ratio 0.44) and E2F transcription factor 3 (gene expression ratio 0.42) were moderately downregulated in the rapamycin plus CN-A treated cells.

### Time course of highly upregulated gene expression by rapamycin plus cotylenin A

**Figure 7:**
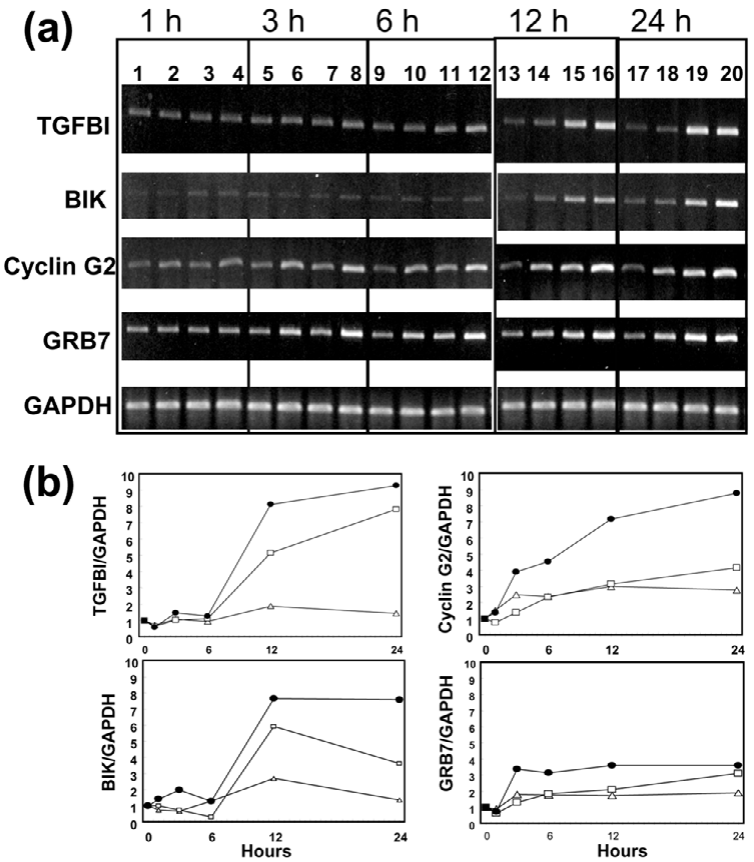
Time courses of TGFBI, BIK, cyclin G_2 _and GRB7 mRNA expression in MCF-7 cells. **(a) **MCF-7 cells were treated without (lanes 1, 5, 9, 13 and 17) or with 0.5 ng/ml rapamycin (lanes 2, 6, 10, 14 and 18), 10 μg/ml CN-A (lanes 3, 7, 11, 15 and 19), or 0.5 ng/ml rapamycin plus 10 μg/ml CN-A (lanes 4, 8, 12, 16 and 20) for 1, 3, 6, 12 and 24 hours. Expression of mRNAs was examined by RT-PCR analysis. **(b) **Graphs depict the relative levels of mRNA after normalization to GAPDH mRNA levels in Fig. 5a. Cells were treated with 0.5 ng/ml rapamycin (white triangle) or 10 μg/ml CN-A (white square), or both combined (black circle). BIK, BCL2-interacting killer; CN-A, cotylenin A; GAPDH, glyceraldehyde-3-phosphate dehydrogenase; GRB, growth factor receptor-bound; TGFBI, transforming growth factor-β-induced 68 kDa protein.

To confirm the results of the cDNA microarrray analysis, the expressions of highly upregulated genes were examined by RT-PCR. Figure 7a shows the time course patterns of gene expression of TGFBI, BIK, cyclin G_2 _and GRB7. Figure 7b shows graphs of the relative levels of mRNA after normalization to GAPDH mRNA levels. These findings confirm the data from the cDNA microarray analysis. In these genes, the expression of cyclin G_2 _was increased fourfold at 3 hours after combined treatment and further increased with time (>8 fold at 24 hours). The expression of GRB7 was also induced by more than threefold at 3 hours after combined treatment, and the expression levels remained almost constant, at least until 24 hours. Expression of the TGFBI and BIK genes were markedly induced by the combined treatment after 12 hours. These genes were quickly and highly upregulated after the addition of rapamycin plus CN-A, and so they may play a crucial role in growth inhibition in human breast carcinoma cells treated with rapamycin plus CN-A.

### Effects of rapamycin and cotylenin A on the *in vivo *growth of MCF-7 cells as xenografts

**Figure 8:**
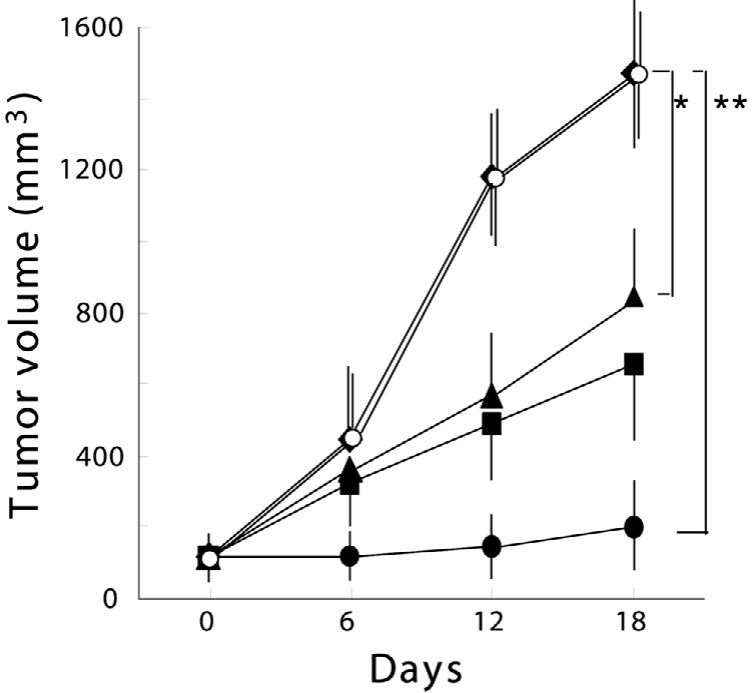
Effects of rapamycin and CN-A on the growth of MCF-7 cells as xenografts. Mice were given a daily intraperitoneal injection of 100 ng rapamycin (black square, black circle) and/or subcutaneous injection of 100 μg of CN-A (black triangle, black circle) every other day. Values are expressed as mean ± standard deviation of 20 mice. Black diamond, untreated mice; white circle, mice treated with solvent alone. CN-A, cotylenin A; *, *p* < 0.05; **, *p* < 0.001.

The *in vitro *studies described above suggested that combined treatment with rapamycin and CN-A should be more therapeutically effective than treatment with rapamycin or CN-A alone. At day 20 after inoculation of human breast carcinoma MCF-7 cells the tumour volume (mean ± standard deviation) was 119 ± 59 mm^3^, and treatments were then started. We administered 0.1 μg rapamycin/mouse every day and 100 μg CN-A/mouse every other day. These treatments had no appreciable adverse effects on the mice. The combined treatment significantly inhibited the growth of MCF-7 cells as xenografts (Fig. 8). Injections of solvent alone did not inhibit the growth of MCF-7 cells *in vivo *(Fig. 8). At day 18 after treatment, the mean tumour volumes in untreated, rapamycin treated, CN-A treated, and rapamycin + CN-A treated nude mice were 1336 ± 332 mm^3^, 659 ± 181 mm^3^, 848 ± 237 mm^3 ^and 245 ± 62 mm^3^, respectively. Although rapamycin and CN-A each significantly retarded tumour growth (*P *< 0.05), combined treatment induced the arrest of tumour growth. The treatment was continued for 18 days and then stopped, with follow up on day 48. All of the untreated, rapamycin treated and CN-A treated mice had a greater tumour burden at day 48. On the other hand, all mice treated with rapamycin plus CN-A still had a much smaller tumour burden (data not shown), suggesting that the therapeutic effects were still maintained after treatment was terminated. These findings indicate that the combination of rapamycin and CN-A is more therapeutically effective than treatment with rapamycin or CN-A alone, and the combined treatment had a significant antitumour effect (*P *< 0.001).

## Discussion

Rapamycin and its analogues are now in clinical trials as anticancer agents that may potently inhibit tumour cell proliferation [4-7]. We have reported that rapamycin or CN-A alone could induce the differentiation of human myeloid leukaemia cells [12-16]. We previously screened the antiproliferative effects of differentiation inducers in myeloid leukaemia cells on several solid tumour cells. In this study, we found that combined treatment with rapamycin and CN-A synergistically inhibited the proliferation of human breast cancer MCF-7 cells *in vitro *and had a marked antitumour effect on MCF-7 cells grown in nude mice. The combined treatment induced growth arrest of the cells at the G_1 _phase rather than apoptosis. Furthermore, the combined treatment strongly enhanced the expression of E-cadherin. CN-A and CN-A plus rapamycin was able to induce SA-βGal activity, which is one of the markers of senescence of epithelial cells [21]. These findings suggest that the combined treatment may induce phenotypic changes toward cell senescence but not apoptosis.

In the present study we showed that the proliferation of human mammary carcinoma MCF-7 cells was synergistically inhibited by treatment with the combination of rapamycin and CN-A. Similar growth-inhibitory effects of rapamycin and CN-A were observed in two other human breast cancer cell lines (oestrogen receptor-positive T-47D cells and oestrogen receptor-negative MDA-MB-231 cells; Fig. 3a,b). These results suggest that the growth-inhibitory activity of rapamycin and CN-A may be independent of the presence of oestrogen receptor in tumour cells. Furthermore, similar growth-inhibitory effects of rapamycin and CN-A were also observed in human non-small-cell lung carcinoma A549 and Lu99 cells (data not shown) and human promyelocytic leukaemia NB-4 cells (Fig. 3c), but not in human monocytic leukaemia THP-1 cells or human ovary carcinoma OVCAR-5 cells. Rapamycin barely inhibited the proliferation of THP-1 cells and OVCAR-5 cells, although CN-A dose dependently inhibited the proliferation of THP-1 cells and OVCAR-5 cells (data not shown). These results indicate that the synergistic growth-inhibitory effects of rapamycin plus CN-A are observed not only in MCF-7 cells but also in other cancer cells, and suggest that moderate to low sensitivity, but not insensitivity, to rapamycin is necessary to exert the synergistic growth-inhibitory effects of rapamycin plus CN-A in cancer cells.

Previous papers have demonstrated that rapamycin induces the inhibition of cell proliferation and G_1 _arrest possibly due to downregulation of the expressions of cyclin D_1 _and c-myc, and upregulation of the expressions of p21^Cip1 ^and p27^Kip1 ^through rapamycin-induced mTOR inhibition in various cancer cells, including MCF-7 cells [5,6]. Furthermore, various agents such as PM-3 (a benzo-γ-pyran derivative), flavopiridol, 2-(4-amino-3-methylphenyl)-5-fluorobenzothiazole (5F-203; an anticancer drug) and oncostatin M induced growth arrest of MCF-7 cells and inhibited the expression of cyclin D_1 _and/or c-myc, or upregulated the expression of p21^Cip1 ^at the transcriptional level [21,29-31]. These reports suggest that downregulation of the expressions of cyclin D_1 _and c-myc, and up-regulation of the expressions of p21^Cip1 ^and p27^Kip1 ^are important to growth arrest of MCF-7 cells.

In the present study, at the doses used, neither rapamycin nor CN-A alone induced G_1 _arrest or significantly affected the expressions of cyclin D, p21^Cip1^, p27^Kip1 ^or c-myc. Furthermore, even when rapamycin in combination with CN-A induced G_1 _arrest of MCF-7 cells, the expressions of these genes were not significantly affected. These findings suggest that other critical genes are important for the induction of growth arrest of MCF-7 cells by rapamycin plus CN-A. Alternatively, the synergistic effects of rapamycin plus CN-A may be exerted by an mTOR-independent pathway. However, this possibility is extremely unlikely because the synergistic effects of rapamycin and CN-A could be blocked by the presence of FK506 on MCF-7 cells (data not shown). It is believed that rapamycin and FK506, which have structural similarities, compete for binding to FKBP12. However, only the complex between rapamycin and FKBP12 is able to bind to mTOR, thereby inhibiting mTOR's role in protein synthesis and the cell cycle [4-7].

Among highly modulated genes induced by rapamycin plus CN-A, we postulate that cyclin G_2_, TGFBI, BIK, GRB7 and EGR3 may play important roles in the induction of the growth inhibition of MCF-7 cells by rapamycin plus CN-A. Cyclin G_2_, together with cyclin G_1 _and cyclin I, defines a novel cyclin family expressed in terminally differentiated tissues. Cyclin G_2 _expression is upregulated as cells undergo cell cycle arrest or apoptosis in response to inhibitory stimuli independent of p53 [25,26,32]. We found a marked induction (>6-fold) in cyclin G_2 _in rapamycin plus CN-A treated MCF-7 cells (Table 2, Fig. 7). The expression of cyclin G_2 _was significantly upregulated at 1 hour after combined treatment and then further upregulated with time (>8-fold at 24 hours; Fig. 7). Frasor and coworkers [33] reported that in oestrogen-treated MCF-7 cells, the expression of cyclin G_2 _was quickly downregulated. Maxwell and coworkers [34] reported a microarray analysis after treatment of MCF-7 cells with 5-fluorouracil, and found that 5-fluorouracil upregulated the expression of cyclin G, although they did not discriminate between cyclin G_1 _and cyclin G_2_. These previous reports and our findings suggest that, in human breast cancer MCF-7 cells, cyclin G_2 _may be a key negative regulator of cell cycle progression.

TGFBI is an extracellular matrix protein whose expression can be induced by transforming growth factor-β [23]. Previous reports [23,35,36] have suggested that TGFBI is involved in cell growth, cell differentiation, cell adhesion and apoptosis, and that it may act as a tumour suppressor. Rapamycin plus CN-A strongly induced TGFBI expression in MCF-7 cells. Because the expression of transforming growth factor-β_3 _was upregulated 2.2-fold in these cells at 12 hours after treatment with rapamycin plus CN-A (microarray data; data not shown), it will be interesting to determine whether the marked induction of TGFBI expression is mediated by the induction of transforming growth factor-β_3 _gene expression.

BIK is a BH3-only proapoptotic protein and forms heterodimers with various antiapoptotic proteins, including Bcl-2 and Bcl-X_L _[24]. BIK triggers apoptosis through a p53-independent pathway. Systemically administrated BIK inhibited the growth of human breast cancer cells implanted in nude mice and prolonged the life span of the treated animals [37]. Although we did not observe evidence of induction of apoptosis in combination-treated MCF-7 cells, upregulated BIK may contribute to the suppression of growth in MCF-7 cells.

GRB7 is an adaptor molecule that mediates signal transduction from multiple cell surface receptors to various downstream signalling pathways. GRB7 and its related family members GRB10 and GRB14 share a conserved molecular architecture including Src homology 2 (SH2) and pleckstrin homology (PH) domains. GRB7 has been implicated to be a downstream mediator of integrin–FAK signal pathways in the regulation of cell migration [27], whereas recent studies have suggested that GRB10 and GRB14 play important roles in cell proliferation [38,39]. In the present study, we found that rapamycin plus CN-A specifically upregulated expression of the GRB7 gene but not the expressions of other genes in this family (Table 2, data not shown). Because the biological roles and molecular mechanisms of this family of genes are still not well understood, it is possible that the specific and early upregulation of GRB7 gene expression may contribute to the combined treatment-induced inhibition of the growth of MCF-7 cells.

EGR3 is an immediate-early and zinc-finger transcription factor [28]. Oestradiol-treated MCF-7 cells exhibited rapid and marked induction of EGR3 gene expression [33,40]. The EGR3 gene is a critical transcription factor for Fas ligand expression in MCF-7 cells as well as T cells [40,41]. Inoue and coworkers [40] suggested that EGR3 plays an important role in the oestrogen-dependent induction of the immune evasion system in oestrogen receptor-positive breast cancer. The present data showed that the expression of EGR3 was markedly downregulated by CN-A alone or rapamycin plus CN-A in MCF-7 cells. Thus, MCF-7 cells in which expression of the EGR3 gene is downregulated might be more susceptible to immune surveillance *in vivo*.

We showed that rapamycin and CN-A cooperatively induced growth arrest in breast carcinoma MCF-7 cells *in vitro*, and that treatment with the combination of rapamycin and CN-A more strongly inhibited the growth of MCF-7 cells as xenografts *in vivo *than did treatment with rapamycin or CN-A alone. The combined treatment also induced the arrest of tumour growth (Fig. 8). Even when treatment was continued for 18 days and then stopped, with follow up on day 48 all of the mice treated with rapamycin plus CN-A still had a much smaller tumour burden (data not shown). These results suggest that the combination of rapamycin and CN-A also induced growth arrest of the cells at the G_1 _phase *in vivo *and then might induce cell senescence. This treatment has no apparent effects on mice (with regard to body weight and behaviour). Taken together, these findings suggest that treatment with the combination of rapamycin and CN-A may be a promising therapeutic strategy for human breast cancer, although the mechanisms underlying the synergistic action of this combined treatment require further investigation.

## Conclusion

We found that rapamycin and CN-A, a novel inducer of the differentiation of myeloid leukaemia cells, synergistically inhibited the proliferation of mammary carcinoma MCF-7 cells. This combined treatment induced growth arrest of the cells at the G_1 _phase, rather than apoptosis, and induced activity of SA-βGal, which is one of the markers of senescence of epithelial cells. Although the expressions of p53, p21^Cip1^, p27^Kip1 ^and cyclin D_1 _mRNAs did not significantly change, we found that expressions of several genes such as cyclin G_2_, TGFBI, BIK, GRB7 and ERG3 were markedly modulated in MCF-7 cells treated with rapamycin plus CN-A. Furthermore, the combined treatment with rapamycin and CN-A significantly inhibited the growth of MCF-7 cells as xenografts without apparent adverse effects, suggesting this combination may have therapeutic value in treating breast cancer.

## Abbreviations

BIK = BCL2-interacting killer; CI = combination index; CN-A = cotylenin A; EGR = early growth response; FKBP12 = FK506-binding protein; GAPDH = glyceraldehyde-3-phosphate dehydrogenase; GRB = growth factor receptor-bound; mTOR = mammalian target of rapamycin; MTT = 3-(4,5-dimethylthiazol-2-yl)-2,5-diphenyltetrazolium bromide; PBS = phosphate-buffered saline; RT-PCR = reverse transcription polymerase chain reaction; SA-βGal = senescence-associated β-galactosidase; TGFBI = transforming growth factor-β-induced 68 kDa protein.

## Competing interests

The authors declare that they have no competing interests.

## Authors' contributions

TK participated in the design of the study and carried out *in vitro *testing and data analysis, and prepared the manuscript. JO-K contributed to its critical revision for important intellectual content. NK and TS participated in the preparation of CN-A and its derivatives. YH contributed to the design of the study and carried out *in vivo *experiments.

## Figures and Tables

**Table 1:** Oligonucleotides used in PCR amplification

	Sense strand	Antisense strand
TGFBI	5'-TGTGTGCTGTGCAGAAGGTT-3'	5'-ATATGGTAGCGGAGGGCATT-3'
BIK	5'-CTCCAGAGACATCTTGATGG-3'	5'-TGGGATCTCCAGAACCTCATT-3'
Cyclin G_2_	5'-AGCACTTGGCAGGTCATGAA-3'	5'-CAACTATTCTAGCAGCCAGC-3'
GRB7	5'-TCTGCCTGAGGAGGTAAAGA-3'	5'-GGAGCTCTTGAACAGTTCGT-3'
EGR3	5'-CTACTTGGGAAAGTTCGCCT-3'	5'-GAATGCCTTGATGGTCTCCA-3'
GAPDH	5'-GGTCGGAGTCAACGGATTTG-3'	5'-ATGAGCCCCAGCCTTCTCCAT-3'

BIK, BCL2-interacting killer; EGR, early growth response; GAPDH, glyceraldehyde-3-phosphate dehydrogenase; GRB, growth factor receptor-bound; TGFBI, transforming growth factor-β-induced 68 kDa

**Table 2:** Upregulated genes in MCF-7 cells after exposure to rapamycin plus CN-A for 12 hours

		Gene expression ratio		
		
GenBank number	Gene name	Rapamycin + CN-A	Rapamycin	CN-A
NM_000358.1	Transforming growth factor-β-induced 68 kDa (TGFBI)	8.16	1.74	5.2
NM_001038.1	Sodium channel, nonvoltage-gated 1α(SCNN1A)	6.99	1.49	4.26
NM_002281.2	Keratin, hair, basic-1 (KRTHB1)	6.96	1.27	12.8^a^
NM_001197.3	BCL2-interacting killer (apoptosis-inducing; BIK)	6.76	2.37	3.59
NM_004354.1	Cyclin G_2 _(CCNG2)	6.08	3.07	3.22
NM_005310.1	Growth factor receptor-bound protein 7 (GRB7)	5.81	2.48	2.61
NM_020898.1	KIAA1536 protein (KIAA1536)	5.42	2.78	1.8
NM_030777.2	Solute carrier family 2, member 10 (SLC2A10)	5.09	1.53	2.51
NM_005727.2	Tetraspan 1 (TSPAN-1)	4.61	1.54	1.76
NM_024709.1	Hypothetical protein FLJ14146 (FLJ14146)	4.55	1.8	2.53
NM_014376.1	Cytoplasmic FMR1 interacting protein 2 (CYFIP2)	4.21	2.43	1.66
NM_017644.1	Hypothetical protein FLJ20059 (FLJ20059)	4.09	1.51	1.2
NM_002166.1	Inhibitor of DNA binding 2 (ID2)	4.03	1.23	2.93
NM_006813.1	Proline rich 2 (PROL2)	3.87	2.8	1.9
NM_002885.1	RAP1, GTPase activating protein 1 (RAP1GA1)	3.81	1.56	2.51
NM_000755.2	Carnitine acetyltransferase (CRAT)	3.79	1.75	2.45
NM_031477.2	Hypothetical protein MGC10500 (MGC10500)	3.72	2.18	2.29
NM_012257.2	HMG-box containing protein 1 (HBP1)	3.7	1.67	1.55
NM_020169.2	Latexin protein (LXN)	3.68	2.49	2.40
NM_014417.2	BCL2 binding component 3 (BBC3)	3.55	1.82	2.18
NM_024642.2	GalNAc-T12 (GALNT12)	3.49	1.49	2.53
NM_016162.1	Inhibitor of growth family, member 4 (ING4)	3.47	1.98	1.73
NM_017673.2	Chromosome 1 open reading frame 26 (C1orf26)	3.45	1.44	2.93
NM_014353.2	RAB26, member RAS oncogene family (RAB26)	3.42	2.40	1.49
NM_005178.2	B-cell CLL/lymphoma 3 (BCL3)	3.39	1.05	3.31

^a^Increased upper reliable levels. CN-A, cotylenin A.

**Table 3:** Downregulated genes in MCF-7 cells after exposure to rapamycin plus CN-A for 12 hours

		Gene expression ratio		
		
GenBank number	Gene name	Rapamycin + CN-A	Rapamycin	CN-A
XM_097230.3	Similar to Cerebellin-like glycoprotein 1 (LOC147381)	0.15	0.93	0.22
NM_006417.2	Interferon-induced protein 44 (IFI44)	0.18	0.62	0.24
XM_095568.5	Hypothetical protein DKFZp762C1112 (DKFZp762C1112)	0.19	1.13	0.29
NM_006820.1	Chromosome 1 open reading frame 29 (C1orf29)	0.20	0.83	0.21
NM_004430.1	Early growth response 3 (EGR3)	0.21	0.53	0.19
NM_024563.1	Hypothetical protein FLJ14054 (FLJ14054)	0.22	0.52	0.45
NM_017816.1	Hypothetical protein FLJ20425 (LYAR)	0.28	0.44	0.45
NM_004934.2	Cadherin 18, type 2 (CDH18)	0.28	1.10	0.20
NM_152454.1	Hypothetical protein FLJ31461 (FLJ31461)	0.29	1.11	0.29
XM_084501.3	Hypothetical protein LOC143381 (LOC143381)	0.29	1.36	0.24
NM_004704.1	RNA, U3 small nucleolar interacting protein 2 (RNU3IP2)	0.30	0.61	0.65
NM_006993.1	Nucleophosmin/nucleoplasmin, 3 (NPM3)	0.32	0.48	0.47
NM_003504.2	CDC45 cell division cycle 45-like (S. cerevisiae) (CDC45L)	0.32	0.48	0.69
XM_059689.2	Similar to agCP15329 (LOC134111)	0.34	0.69	0.38
NM_015179.1	KIAA0690 protein (KIAA0690)	0.35	0.56	0.71
NM_014520.1	MYB binding protein (P160) 1a (MYBBP1A)	0.35	0.53	0.62
XM_300777.1	Similar to muscle protein (LOC348523)	0.35	0.70	0.69
NM_032509.1	RNA binding protein (LOC84549)	0.35	0.47	0.75
NM_014503.1	Downregulated in metastasis (DRIM)	0.36	0.48	0.56
NM_000269.1	Nonmetastatic cells 1, protein (NM23A) expressed in (NME1)	0.37	0.58	0.65
XM_050219.6	Synaptopodin 2 (SYNPO2)	0.37	0.93	0.57
XM_295865.1	LOC340895 (LOC340895)	0.37	1.34	0.33
NM_133263.1	PPAR gamma coactivator 1β(PERC)	0.37	0.62	0.74
NM_002220.1	Inositol 1,4,5-trisphosphate 3-kinase A (ITPKA)	0.37	0.60	0.62
NM_015068.1	Oaternally expressed 10 (PEG10)	0.38	0.77	0.43

CN-A, cotylenin A.

**Table 4:** Effects of rapamycin plus CN-A on cell cycle regulatory genes

	Gene expression ratio		
	
Gene name	Rapamycin + CN-A	Rapamycin	CN-A
Cyclin A_2_	0.51	0.54	0.82
Cyclin B_1_	0.71	0.77	0.84
Cyclin B_2_	0.84	0.89	0.89
Cyclin D_1_	0.94	0.96	1
Cyclin D_3_	0.86	0.7	1.09
Cyclin E_1_	0.44	0.67	0.66
Cyclin E_2_	0.53	0.64	0.87
Cyclin G_1_	1.43	1.25	1.02
Cyclin G_2_	6.08	3.07	3.22
			
Cyclin-dependent kinase 2 (CDK2)	0.58	0.78	0.83
Cyclin-dependent kinase 4 (CDK4)	0.62	0.81	0.77
Cell division cycle 2 (CDC2)	0.62	0.7	0.85
			
P53	1.37	1.52	1.32
P21 (Cip1)	0.85	0.83	1.03
P27 (Kip1)	1.65	1.39	1.18
			
Retinoblastoma 1 (RB1)	1.08	1.09	1.04
Retinoblastoma-like 1 (p107)	0.53	0.67	0.87
Retinoblastoma-like 2 (p130)	1.96	2.18	1.07
E2F transcription factor 3 (E2F3)	0.42	0.86	
E2F transcription factor 5, p130-binding (E2F5)	0.96	1.21	0.81
E2F transcription factor 6 (E2F6)	0.62	0.70	0.65

CN-A, cotylenin A.
